# Diet Quality and Water Scarcity: Evidence from a Large Australian Population Health Survey

**DOI:** 10.3390/nu11081846

**Published:** 2019-08-09

**Authors:** Bradley G. Ridoutt, Danielle Baird, Kimberley Anastasiou, Gilly A. Hendrie

**Affiliations:** 1 Commonwealth Scientific and Industrial Research Organisation (CSIRO) Agriculture and Food, Clayton South, VIC 3168, Australia; 2 Department of Agricultural Economics, University of the Free State, Bloemfontein 9300, South Africa; 3 CSIRO Health and Biosecurity, Adelaide, SA 5000, Australia

**Keywords:** dietary guidelines, discretionary food, life cycle assessment, sustainable diet, sustainable food production, sustainable food systems, water footprint, water use

## Abstract

There is widespread interest in dietary strategies that lower environmental impacts. However, various forms of malnutrition are also widely prevalent. In a first study of its kind, we quantify the water-scarcity footprint and diet quality score of a large (>9000) population of self-selected adult daily diets. Here, we show that excessive consumption of discretionary foods—i.e., energy-dense and nutrient-poor foods high in saturated fat, added sugars and salt, and alcohol—contributes up to 36% of the water-scarcity impacts and is the primary factor differentiating healthier diets with lower water-scarcity footprint from poorer quality diets with higher water-scarcity footprint. For core food groups (fruits, vegetables, etc.), large differences in water-scarcity footprint existed between individual foods, making difficult the amendment of dietary guidelines for water-scarcity impact reduction. Very large reductions in dietary water-scarcity footprint are possible, but likely best achieved though technological change, product reformulation and procurement strategies in the agricultural and food industries.

## 1. Introduction

Water scarcity is a major global environmental concern [1,2], identified in Target 6.4 of the United Nations Sustainable Development Goals (SDGs) [3]. The scarcity of freshwater impacts the health of freshwater and estuarine ecosystems [4], is a threat to human hygiene, sanitation [5,6] and food security [7,8], and can have serious negative implications for economic development [9,10]. Future conflicts over limited water resources have even been foreshadowed [11]. While water scarcity varies greatly from one geographic region to another [12,13], and along with this the importance of local action to limit water consumption, the interconnected nature of the world economic system means that the problem of water scarcity is shared globally.

One approach to addressing the water scarcity problem is to increase water-use efficiency, particularly in the water-intensive sectors of the economy, and this is emphasized in Sustainable Development Goals (SDG) Target 6.4. However, the need to transition toward sustainable consumption patterns, expressed in SDG 12, is also important, and in this regard the food system is critically relevant, accounting for around 70% of global freshwater use [14]. As such, an interest has emerged to identify and recommend dietary strategies that have lower environmental impacts from water use [15,16,17,18,19], as part of a movement toward sustainable diets [20,21,22]. That said, SDG 2, which aims to end all forms of hunger and malnutrition by 2030, is also critically relevant. Dietary strategies to lower environmental impacts must also be nutritionally adequate [23].

The state of knowledge concerning dietary patterns and how they contribute differently to water scarcity is currently rather limited. The sustainable diets literature is dominated by studies addressing the greenhouse gas emissions of diets, and when water is considered it is usually water use that is assessed, not water scarcity [15]. Diets are composed of many different food products, each with its own product life cycle. Water use can occur in regions of water scarcity and in regions of water abundance and when water use in different environmental settings is aggregated, the results become unintelligible [24,25,26]. This is why the international water footprint standard, ISO14046:2014 [27], forbids such practice and requires application of a water-scarcity impact assessment model in the calculation of a water footprint. Another limitation of the current evidence base concerning diets and water scarcity is that assessments have been of average diets or dietary scenarios, not actual individual diets [28,29,30,31]. Actual diets can include a high content of discretionary foods which lead to excess energy consumption, inadequate intake of micronutrients, and inflated environmental impacts [32,33,34].

In this study, we use life-cycle assessment (LCA) to model the water-scarcity footprint of 9341 individual adult Australian daily diets obtained by a structured 24-h recall process as part of the Australian Health Survey [35]. In addition, a diet quality score was calculated, enabling the identification of actual diets in the Australian community characterized by higher diet quality and lower water-scarcity footprint. Our objective was to contribute evidence in support of future revision of food-based dietary guidelines and identify intervention strategies in support of sustainable production and consumption of food. To our knowledge this is the first study to report the water-scarcity footprint for a large number of individual self-selected diets.

## 2. Methods and Data

### 2.1. Dietary Intake Data

The 2011–2013 Australian Health Survey is the most recent and most comprehensive population health survey conducted in Australia by the Australian Bureau of Statistics. The Survey is nationally representative and comprises three components, one being the National Nutrition and Physical Activity Survey [35]. Detailed dietary intake data were collected from 12,153 participants, and this study of diet quality and water scarcity utilizes information from the 9341 adults who were selected to participate from across Australia. The complex sampling method and design of the survey [36] mean that estimates of dietary intake for the Australian population as well as demographic subgroups can be made using weights that indicate how many population units are reflected by each sample unit.

Dietary intake data were collected over a 13-month period using a 24-h recall process, whereby participants describe all foods and beverages and portion sizes consumed on the day prior to an interview that was undertaken face-to-face with a trained interviewer. To allow food and nutrient intakes to be estimated from the dietary intake data, the Australian Bureau of Statistics uses a classification system that groups similar foods together. As such, each of the 5645 individual foods recorded in the survey has an 8-digit identification code assigned. This coding is based on a three-tiered structure, whereby the first two digits refer to the major food group the food belongs to, based on its key ingredient. There are 24 major food groups, which are then broken down into 132 sub-major food groups and around 500 minor groups.

To enable the integration of the dietary intake data with the water-scarcity footprint data, each of the 5645 individual foods at the 8-digit level, many of which were processed foods made from multiple ingredients or mixed dishes, were disaggregated into basic components. For example, an apple pie was disaggregated into apple, sugar, flour and shortening as major components. This was undertaken using recipe files published by the Australian Bureau of Statistics as part of the National Nutrition and Physical Activity Survey [35], supplemented by ingredient information available on food packaging and food manufacturers’ websites. The same approach was taken to foods of all ethnic origins. To translate cooked food portions into raw quantities, conversion factors were applied that were obtained predominantly from a reference database maintained by Food Standards Australia and New Zealand [37]. For example, cooked meat typically loses moisture during cooking, and a factor of 1.3 was applied. Rice absorbs moisture during cooking in the ratio of 2 parts water to 1 part grain. These adjustment factors were applied without impact on the nutrient profile of the foods.

A common feature of dietary intake surveys is the possibility that participants did not always recall accurately, or chose to deliberately under-report, the foods and portion sizes eaten. In order to assist in the interpretation of the nutrition survey data, the Australian Bureau of Statistics has produced estimates of the under-reporting prevalence. These factors (21% for females and 17% for males) were uniformly applied to all dietary intake data. While it is possible that under-reporting was biased toward certain types of foods (e.g., discretionary foods) and individuals of particular weight status, insufficient evidence exists to support a method involving more specific allocation of under-reported food energy. The correction for under-reporting is necessary to allow comparison between current reported diets and recommended diets (which are not impacted by under-reporting).

After application of the under-reporting factor, dietary intake was determined for each of the 9341 Australian adult diets. Dietary intake was expressed in terms of energy intake (kilojoules) and number of serves of each of the food groups described in the official Australian Dietary Guidelines [38]: e.g., fruits, vegetables, grain (cereal) foods, meats and alternatives, dairy products and alternatives, discretionary foods. Beverages, such as tea and coffee, which were not included in the aforementioned food groups formed a separate category. Within the category meat and alternatives, we also examined red meat (beef and lamb), pork, poultry, seafood, vegetarian alternatives (eggs, tofu and nuts) and other native meats and offal. This was due to the particular focus on the contribution of meat to dietary water footprints appearing in the literature [39,40,41]. Due to the public health nutrition concerns about excessive consumption of discretionary foods, we also examined various sub-categories of discretionary foods.

The mean dietary intake for Australian adults was also calculated by applying the population weighting factors (described above). In addition, an adjustment factor was applied to account for the uneven representation of data across days of the week, as food habits can vary throughout the week, and especially on weekends compared to weekdays. Mean values were expressed for age and gender subgroups (19 to 50 years, 51 to 70 years and 71 years and above) corresponding with the Australian Dietary Guidelines.

### 2.2. Diet Quality Analysis

Diet quality was assessed using the food-based Dietary Guideline Index of Golley et al. [42] with the scoring adapted to the dietary intake targets described for adults in the Australian Dietary Guidelines [38]. The Diet Quality Index reflects overall compliance with the guidelines in terms of the amount and quality of food consumed from the core food groups, discretionary foods and beverages, as well as diet variety. The index comprises 11 components and individuals receive a diet quality score out of 100, where a higher score reflects greater compliance with the Guidelines.

### 2.3. Water-Scarcity Footprint Modelling

Water-scarcity footprint modelling was undertaken using a hybrid life-cycle assessment (LCA) approach combining economic input-output LCA and process-based LCA. For the main agricultural commodities, water-scarcity footprint data were obtained from a previous study [43], where a spatially disaggregated water use account and spatially-explicit water-scarcity factors were used to develop water-scarcity extensions that were coupled with a multi-regional input–output model. The results link demand for agricultural commodities in Australia to the problem of water scarcity in Australia and globally. Australia is a net exporter of most agricultural commodities [44]. It has been estimated that more than 90% of food consumed domestically is currently grown in Australia [45]. For agricultural commodities not typically grown in Australia—tea leaves, coffee beans, hops, palm fruit, coconut, cocoa and hazelnut—water-scarcity footprints were quantified using agricultural water use (consumption) data for the major regions supplying Australia [46] taken from a study by Pfister et al. [47] (see Supplementary Table S4 for more detail). For seafood caught in the wild and aquaculture production, water-scarcity footprints were quantified using water use data taken from literature [48,49,50,51] combined with a spatially-disaggregated description of the number of Australian businesses in the fishing and aquaculture industry sectors obtained from the Australian Bureau of Statistics [52]. The water-scarcity footprint of aquaculture seafood included feed inputs (see Supplementary Table S5 for more detail). It is noted that water-scarcity footprints of commodities sourced outside Australia and of seafood products are not as comprehensive in their coverage of supply chain water use as those of Australian agricultural commodities. However, water use in the production of farming inputs, such as fuels, fertilizers, etc., generally makes only a modest additional contribution to the water-scarcity footprints of agricultural commodities [24].

The water-scarcity footprint results for agricultural commodities were used to calculate the water-scarcity footprints for individual foods using a process-based LCA approach using data obtained from literature (see Supplementary Material for details). Conversion factors were used to translate agricultural products into retail products and edible portions. The water scarcity footprints associated with various food processing operations were quantified using water use estimates relevant to each processing industry combined with a spatially-disaggregated description of the number of businesses in each industry in each region of Australia (e.g., meat and meat product manufacturing, seafood processing, dairy product manufacturing, fruit and vegetable processing, etc.). This approach is relevant to a national study of dietary habits where individuals purchase food from a national food system, typically without knowledge about where it was produced.

To calculate the water-scarcity footprint of each food product, water use (consumption) in each spatial unit was multiplied by the relevant water-scarcity factor and summed across the product life cycle. In this study, three sets of spatially explicit water-scarcity factors were applied. ISO14046:2014 describes general requirements for the quantification of a water-scarcity footprint. However, no particular water-scarcity model is prescribed [27]. We chose three different water-scarcity models with distinctly different conceptual bases and model structures. Firstly, a modified form of the Water Stress Index of Pfister et al. [53] was used, whereby individual factors are scaled such that results are presented relative to water use at the global average level of water scarcity [24]. This model, termed World-eq, is based on the ratio of water withdrawal to availability in each region. It has been applied in many water-scarcity footprint studies internationally and is currently recommended by the Australian Life-Cycle Assessment Society [54]. The second water scarcity model applied was the AWARE model [12], chosen because it is the product of an important international collaboration known as the Life-Cycle Initiative (www.lifecycleinitiative.org). The AWARE model is based on an estimate of the relative level of water remaining in each region. The third model integrates results from models that separately assess the impacts of water use in each region on human health and ecosystem quality [55]. Detailed information about the water scarcity models is available in the associated references.

For processed foods combining multiple ingredients, archetypal recipes were used to calculate water-scarcity footprints. The water-scarcity footprints for almost 150 separate food items are presented in the Supplementary Material.

### 2.4. Modelling of Dietary Pattern Scenarios

A quadrant analysis was undertaken by ranking individual daily diets (subgroup of 19- to 50-year-olds) according to water-scarcity footprint and diet quality score. Excluding those daily diets within 0.25 standard deviations of the mean for each parameter, we specifically compared daily diets higher in diet quality and lower in water-scarcity footprint (“best diets”; *N* = 683) and those lower in diet quality and higher in water-scarcity footprint (“worst diets”; *N* = 538). Comparison was also made between the average 19- to 50-year-old adult daily diet and a diet complying with the recommended number of serves of each food group as described in the Australian Dietary Guidelines [38]. The water-scarcity footprint of the recommended diet was calculated using the water-scarcity footprint intensity (L-eq serve^−1^ for each food group) of the average diet and of the “best diet”. This approach avoided the need to make assumptions about the specific foods that were included within the recommended diet. The Australian Dietary Guidelines are not prescriptive about specific food choices within a food group, apart from emphasizing wholegrains over refined grains, whole fruit over juice, and the like. Instead, the guidelines emphasize choosing a variety of foods. Therefore, it is possible to construct very many specific diets complying with the Guidelines.

### 2.5. Other Descriptive Statistics

The Pearson correlation coefficient (*N* = 9341) was used to elaborate the degree of relationship between water-scarcity footprint results obtained using the three water scarcity models. To investigate the extent that variation in dietary energy intake explained variation in dietary water-scarcity footprint, the coefficient of determination was calculated (*N* = 9341). The Pearson correlation coefficient was also used to explore the linear relationship between dietary greenhouse gas (GHG) emissions intensity and dietary water-scarcity footprint intensity (*N* = 9341). Dietary GHG emissions data were obtained from the study of Hendrie et al. [32].

## 3. Results

### 3.1. Water-Scarcity Footprint of Adult Diets

Three different water-scarcity indicators, with distinctly different conceptual bases and model structures, were used to quantify water-scarcity footprints of Australian adult daily diets. The results obtained were very highly correlated (Pearson correlation 0.937 to 0.971, *N* = 9341), consistent with findings reported elsewhere [43,56]. As such, results obtained using the more commonly used World-eq model are presented (see Methods for description). Other results are presented in the Supplementary Material. Using the World-eq model, water-scarcity-footprint results are expressed relative to water use at the global average level of water scarcity to enable international comparison.

The water-scarcity footprints of Australian adult daily diets were highly variable (s.d. 218 L-eq person^−1^ day^−1^), reflecting the great diversity of eating habits in the general community, but averaged 362 L-eq person^−1^ day^−1^. Real diets are composed of very many individual food items, involving water use across a diversity of environmental contexts, some locations higher in water scarcity and others lower in water scarcity. The application of a water-scarcity model in the quantification of a water-scarcity footprint, enables water use in locations of varying water scarcity to be aggregated and the overall contribution to the water scarcity problem expressed. This is akin to the application of global warming potentials to the emissions of different greenhouse gases in the quantification of a carbon footprint. Our results suggest that the average Australian adult daily diet contributes to the global water scarcity problem at a level equivalent to 362 L of water use at the global average level of water scarcity.

There exists a paucity of studies reporting comparable results. Hess et al. reported a water-scarcity footprint for the average UK diet of around 135 L-eq person^−1^ day^−1^ using that same water-scarcity model [28]. This result is less than half the value reported for Australian adult diets. However, the UK study was for the entire UK population (not only adults) and was based on water use in the production of agricultural commodities only, rather than food as consumed. It is unclear the extent to which the lower average dietary water-scarcity footprint reported for the UK was the result of these factors, or the lower average levels of water scarcity across the UK compared to Australia, or differences in dietary intake. Other studies reporting water-scarcity footprint results for diets have either reported comparative results only or have not described the particular water-scarcity model used [30,31]. It has previously been demonstrated that water-scarcity footprint results calculated with different water-scarcity models are not directly comparable due to scaling differences [43,56].

**Figure 1:**
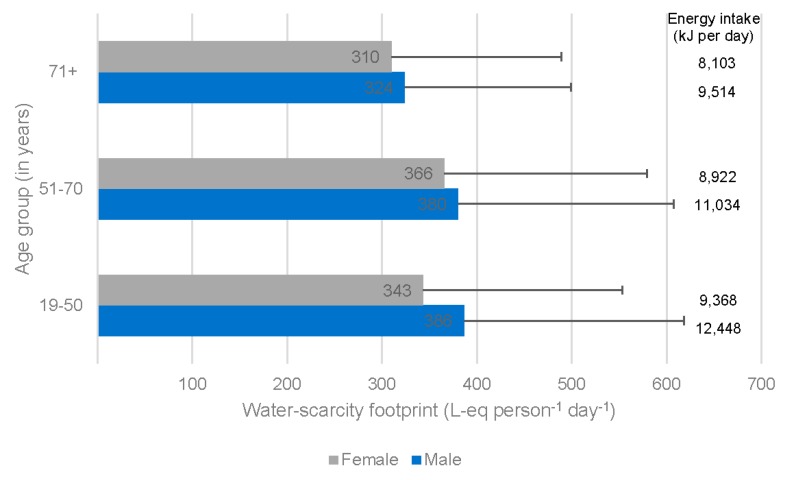
Water-scarcity footprint of Australian adult diets. Results are based on 9341 individual daily diets reported in the Australian Health Survey and are shown separately for females and males and for three age groups. Bars indicate the standard deviation. The dietary energy intake is also shown.

In the Australian community, water-scarcity footprint results were marginally lower for women than for men (346 and 379 L-eq person^−1^ day^−1^, respectively) and for older adults (71 years and above; 316 L-eq person^−1^ day^−1^) than other adults (19–50 years and 51 to 70 years; 365 and 373 L-eq person^−1^ day^−1^, respectively) (Figure 1). To some extent, this reflects differences in overall dietary intake between these sub-groups. Variation in dietary energy intake was found to explain up to one third of the variation in dietary water-scarcity footprint (*R*^2^: Females = 0.33, Males = 0.30).

### 3.2. Contribution Analysis

The foods that contributed most to the total water-scarcity footprint of Australian adult diets were discretionary foods, at around 25% (Table 1). Discretionary foods, also known as non-core foods, are energy-dense and nutrient-poor foods high in saturated fat, added sugars and salt, and alcohol [38]. These foods are readily available in the Australian community and collectively contribute more than 30% of adult dietary energy intake [35]. They can come from predominantly plant origins (e.g., cakes, biscuits, sugar-sweetened beverages, beer, wine) or animal origins (e.g., dairy desserts, processed meats) and frequently combine ingredients from both. Although widely enjoyed, these foods are not a necessary part of a healthy diet. They contribute to excess energy intake linked to weight gain and escalating rates of overweight and obesity [57]. They can also displace nutrient-rich core foods in the diet, thereby contributing to malnutrition in the form of inadequate intake of micronutrients [57]. Previously, it has been shown that discretionary foods contribute around 30% of dietary greenhouse gas emissions in Australia [32].

The next highest contribution to the water-scarcity footprint of Australian adult diets came from fruits (Table 1). This food group includes whole fruit as well as freshly squeezed and commercially prepared fruit juices. Both are considered core foods under the Australian Dietary Guidelines, although it is recommended to mainly consume whole fruit, and juice should only be consumed occasionally and in smaller amounts [38]. This food group does not include sugar sweetened fruit juice drinks that may contain only a fraction of juice, which were included in discretionary beverages. Collectively, fruits and vegetables contributed around 25% to the water-scarcity footprint. Other studies have also drawn attention to the important water-scarcity impacts of fruits and vegetables. In the UK, fruits and vegetables accounted for more than 30% of the water-scarcity footprint of the average diet and this increased marginally under various healthier eating scenarios [28]. In Denmark, vegetarian and vegan diets had, respectively, 11% and 22% higher water-scarcity footprints than the average diet [31]. This is of some concern since fruits and vegetables are nutritionally important food groups that are under consumed in most diets relative to dietary guidelines [58].

Another important food group was dairy products and alternatives (such as those made from legumes, cereals or nuts, e.g., soy beverage, rice beverage, almond beverage), collectively contributing around 16% to the water-scarcity footprint. Fresh meats (i.e., meats that have not undergone any preserving process) and alternatives (such as eggs, tofu and other vegetarian and vegan substitutes) contributed slightly more than 10%. Beverages, including tea and coffee, contributed about 7%. These findings challenge the common perception that meat consumption is the critical factor driving dietary water footprints [16,39,40,41,59]. Here, fresh meats (seafood, beef and lamb, poultry and pork) contributed less than 8% of the total dietary water footprint, and processed meats an additional 5% (Table 1). Most of the evidence supporting this assertion comes from studies where water scarcity impact modelling has not been undertaken. In some cases, the use of natural rainfall by agricultural fields is combined with water abstracted from ground and surface water bodies, effectively precluding any interpretation of results in relation to water scarcity, which is the environmental concern identified in SDG 6.4. Food systems vary greatly from one region of the world to another in their use of irrigation, water use efficiency and intersection with areas of high water scarcity. Therefore, it is difficult to make generalizations about meat consumption and water scarcity. However, in the Australian context, the evidence points to fresh meat consumption being of lesser importance to water scarcity than most other food groups, even cereals.

### 3.3. Water Scarcity and Diet Quality

**Figure 2:**
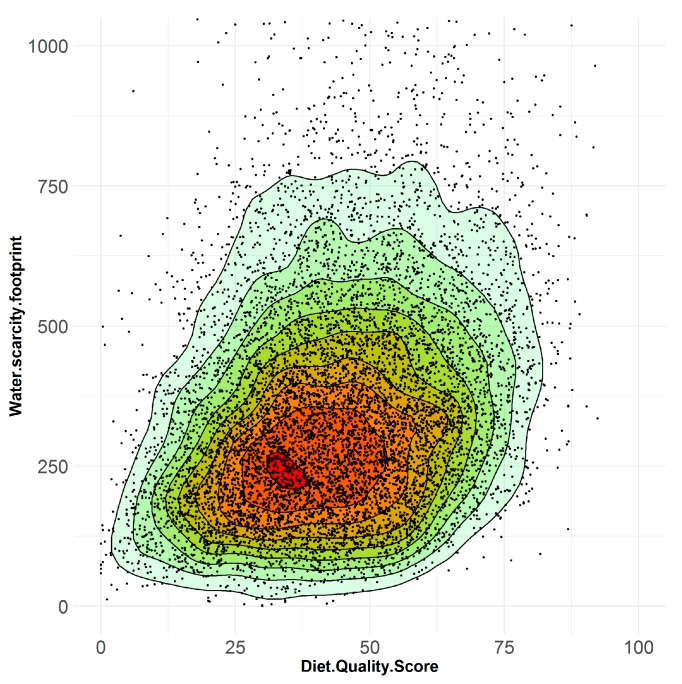
Water-scarcity footprint and diet quality score. Scatterplot showing diversity of individual adult daily diets reported in the Australian Health Survey (*N* = 9341). Healthier diets (with higher diet quality score) do not necessarily have a lower water-scarcity footprint (L-eq person^−1^ day^−1^).

Self-reported adult daily diets in Australia were found to vary widely in both diet quality and water-scarcity footprint (Figure 2). Here, diet quality was estimated using the Dietary Guideline Index [42] which reflects overall compliance with the Australian Dietary Guidelines [38] in terms of the amount and quality of food consumed from the core food groups, discretionary foods and beverages, as well as dietary diversity. The index comprises 11 components and individuals receive a diet quality score out of 100, where a higher score reflects greater compliance with the guidelines.

In Australia, dietary guidelines differ for the different adult life stages (19–50 years, 51–70 years, 71 years and older). For the largest adult subgroup (19–50 years; *N* = 5157), we compared dietary patterns characterized by higher diet quality and lower water-scarcity footprint (HDQ-LWF) to those characterized by lower diet quality and higher water-scarcity footprint (LDQ-HWF) (see Methods for details of quadrant analysis). It is relevant to understand the food choices that differ between these subgroups. Self-reported diets are actual daily diets (in contrast to theoretical dietary scenarios) and it is realistic that more Australians could adopt a dietary pattern similar to the many Australians who are already consuming a healthier and lower water-scarcity footprint diet.

Individuals in the HDQ-LWF subgroup had a 64% lower dietary water-scarcity footprint than individuals in the LDQ-HWF subgroup (207 L-eq person^−1^ day^−1^ compared to 573 L-eq person^−1^ day^−1^, Table 2). The average diet quality score was also twice as high (57.1 compared to 28.4). The overwhelming difference between the two dietary patterns was the intake of discretionary foods. The HDQ-LWF dietary pattern contained 2.37 serves of discretionary food per day contributing a water-scarcity footprint of 23.9 L-eq per day. This is equivalent to consuming a tablespoon of jam or honey at breakfast (1 serving), around 30 g of plain cake or cake-type muffin as a morning or afternoon snack (0.75 serving) and one small glass of wine at dinner (125 mL, 0.625 serving). In sharp contrast, the LDQ-HWF dietary pattern contained more than five times the content of discretionary food (12.3 servings), contributing a water-scarcity footprint of over 200 L-eq per day (Table 2).

The differences between the HDQ-LWF and LDQ-HWF dietary patterns were smaller for all of the core food groups. On average, individuals in the HDQ-LWF subgroup consumed a greater number of serves of fruits and vegetables (grouped together) and more fresh meat (or meat alternatives), but fewer serves of bread and cereals, and of dairy products (or alternatives) (Table 2). The results for meat consumption are especially notable because of the aforementioned prevailing argument that intake of meat is detrimental to both health and dietary water footprint [39,41,59]. The Australian Dietary Guidelines [38] regard lean meat as a core food and suggest that while some men could benefit from eating less red meat, generally, Australians need to eat more lean meats and poultry, fish, eggs, nuts and seeds and legumes/beans (p. 3). In particular, the guidelines suggest that some children and young women could benefit from the additional nutrients associated with an increase in lean red meat consumption (p. 21). Considering dietary water-scarcity footprints, in the Australian context, higher fresh meat consumption (2.66 serves person^−1^ day^−1^ compared to 1.80 serves person^−1^ day^−1^) was associated with a lower dietary water-scarcity footprint (Table 2).

### 3.4. Recommended Dietary Scenario

In comparing the average daily diet of 19- to 50-year-old Australian adults to the recommended diet described in the Australian Dietary Guidelines [38], it is evident that Australians should, on average, increase their intake of all of the five core food groups (Table 3). Most notably, the intake of vegetables should more than double, from 2.47 serves per day to a minimum of 5.5 serves per day (5.0 and 6.0 serves respectively for women and men). In addition, Australian adults should substantially reduce their intake of discretionary foods from the present average of 7.42 serves per day to less than 2.8 (2.5 and 3.0 serves respectively for women and men).

A shift from the current average adult daily diet to the recommended intake of core and discretionary foods has the potential to either increase or decrease the dietary water-scarcity footprint. If the water-scarcity footprint intensity (L-eq serve^−1^) of each food group remained the same as for the current diet, a shift to the recommended diet would see the water-scarcity footprint increase by more than 20%, from 365 to 445 L-eq person^−1^ day^−1^ (Table 3), with the largest increases associated with higher consumption of fruit and vegetables (62.5 L-eq person^−1^ day^−1^) and dairy products and alternatives (39.7 L-eq person^−1^ day^−1^). The decreased consumption of discretionary foods would save slightly more than 50 L-eq person^−1^ day^−1^. However, if the shift to a recommended diet involved food choices with the same water-scarcity footprint intensity as the HDQ-LWF dietary pattern (described above), the overall water-scarcity footprint would instead be lowered by 13% to about 320 L-eq person^−1^ day^−1^ (Table 3).

**Figure 3:**
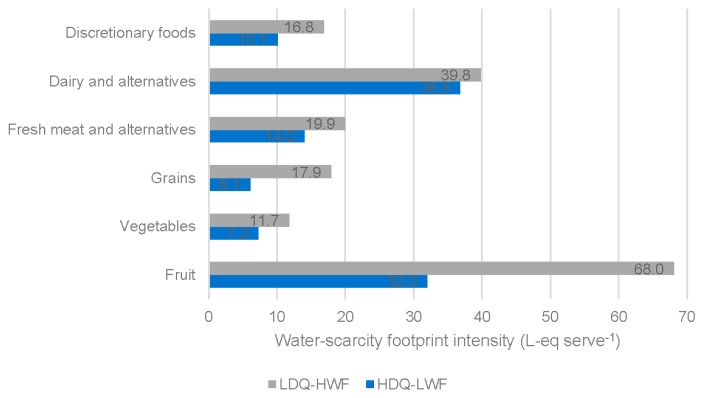
Water-scarcity footprint intensity of major food groups. There exists large differences in water-scarcity footprint intensity between individual foods within some food groups, particularly fruits and grains. Shown here are average results for the higher diet quality and lower water-scarcity footprint (HDQ-LWF) dietary pattern and the lower diet quality and higher water-scarcity footprint (LDQ-HWF) dietary pattern.

It is apparent that the type of food chosen within a food category is an important factor in determining dietary water-scarcity footprints. Figure 3 presents the water-scarcity footprint intensity (L-eq serve^−1^) of major food groups for the HDQ-LWF and LDQ-HWF dietary patterns, where particularly large differences were observed for fruits and for grains. In the case of fruits, a medium sized apple (100 g) with edible portion of 92% has a water-scarcity footprint of 3.0 L-eq. In contrast, 250 mL of freshly squeezed orange juice has a water-scarcity footprint of slightly more than 100 L-eq. It is notable that juice made up 46% of the fruit intake of the LDQ-HWF dietary pattern compared to 28% for the HDQ-LWF dietary pattern. In the case of cereals, similarly large variations in the water-scarcity footprint were found. One cup of cooked rice (67 g uncooked rice) has a water-scarcity footprint of 124 L-eq. This compared to 0.9 L-eq for 2 slices (80 g) of wholegrain bread. Again, it is notable that rice represented 32% of cereal intake in the LDQ-HWF dietary pattern, compared to only 9% for the HDQ-LWF dietary pattern. In the case of meats and meat alternatives, the variation in water-scarcity footprints were smaller. The water-scarcity footprints of a serving of lamb, pork, poultry, beef, egg and tofu were, respectively, 5.5 L-eq (65 g cooked boneless lamb meat, 90 g raw), 8.5 L-eq (80 g cooked boneless chicken meat, 100 g raw), 9.5 L-eq (65 g cooked boneless pig meat, 90 g raw), 11.8 L-eq (65 g cooked boneless bovine meat, 90 g raw), 13.6 L-eq (2 large eggs, 120 g, 107 g excluding shell), 20.0 L-eq (170 g tofu). A glass of wine (125 mL) had a water-scarcity footprint of 41 L-eq. The water scarcity footprint of a 375 mL serving of beer was lower (2.9 L-eq). The water-scarcity footprint of a glass of rum and cola (30 mL rum, 220 mL cola) was lower again (1.7 L-eq). A detailed listing of water-scarcity footprints of individual foods is presented in the Supplementary Material.

## 4. Discussion

### 4.1. Implications for Strategic Action

The objective of reducing water scarcity impacts of the food system through dietary change needs to be considered in the context of water scarcity being only one among several important environmental impacts associated with food production and consumption [60]. A major limitation of footprint studies is their focus upon a single environmental aspect, in contrast to life-cycle assessment which is oriented toward comprehensive assessment of all relevant environmental impacts and evaluation of trade-offs [61]. For this large dataset of 9341 Australian adult daily diets, we found no relationship between dietary GHG emissions, previously reported [32], and water-scarcity footprint after controlling for dietary energy content (Pearson correlation 0.03 to 0.05 depending on the water-scarcity indicator used). We controlled for energy content because dietary GHG emissions and water-scarcity footprint are both correlated with total energy content as explained previously and elsewhere [32]. Therefore, dietary strategies to reduce water-scarcity footprints should have no impact on dietary GHG emissions at the population level, although impacts on other environmental aspects are possible, as noted by Springmann et al. [62].

This study has demonstrated that very large reductions in dietary water-scarcity footprint are possible; however the opportunities to intervene through amended dietary guidelines are not straightforward because there are large variations in water-scarcity footprint intensity between foods within a food group. Diversity is an important principle in nutrition [63]. The Australian Dietary Guidelines emphasize enjoying a wide variety of nutritious foods and eating vegetables of different types and colors [38]. Any guidance to avoid higher water-scarcity footprint fruits, vegetables, grains or protein sources could undermine existing public health nutrition goals. In any case, consumers could not easily identify specific higher water-scarcity footprint foods without individual food labelling, akin to the carbon footprint labelling of foods in some markets. Furthermore, foods having a higher water-scarcity footprint intensity are not necessarily the same as those with higher GHG emissions intensity, so consumers could be faced with making their own trade-off which could be problematic. Sustainable healthy diets need to emphasize improved nutrition as well as lower environmental impacts. Reducing discretionary food and beverage intake would seem to be the common denominator as these foods are consumed in disproportionate quantities in Australia and elsewhere, contributing to excessive food energy consumption which is detrimental to health and has the effect of inflating dietary environmental impacts. The other obvious guideline is to reduce food waste which would lead to a lowering of all forms of resource use and environmental burden in the food system.

Perhaps a more effective approach to reducing water scarcity impacts associated with the food system is to focus on improvements in food production. This might be through technological innovation. For example, Page et al. [64] evaluated the water-scarcity footprint of fresh tomatoes supplied to the Sydney market and reported results ranging from 5.0 to 52.8 L-eq kg^−1^ for a variety of open field, low-technology and high-technology greenhouses, a tenfold difference. Though not addressing water scarcity, Springmann et al. [17] showed that technological changes were more effective than dietary change in reducing food system water use. In the food-processing industry important opportunities also exist to reduce water-scarcity impacts though product formulation and ingredient procurement. For example, the water-scarcity footprint of milk produced in different regions of south-eastern Australia was found to range from 0.7 to 262 L-eq per L [56]. Food manufacturers using dairy ingredients could selectively procure from lower water-scarcity footprint regions. These selected examples highlight the vast scope for water-scarcity footprint reduction across the food production system. In future research it will be important to assess the variation in water-scarcity footprint of different food production systems and undertake further trade analysis.

### 4.2. Limitations

Care was taken to select the highest quality input data at each stage in the analysis. The study utilized dietary intake data from the Australian Health Survey. The very large sample (>9000 adults) and systematic data collection process mean that this dataset can be considered to be of very high quality and representative of the Australian population. However, the under-reporting phenomena, characteristic of all 24-h dietary recall surveys, was a limitation. As discretionary foods are more likely to be under-reported, it is possible that our study has underestimated the actual contribution of these foods to the water-scarcity footprint. It is also acknowledged that 24-h dietary recall surveys do not account for day-to-day variation in eating patterns of individuals.

Water scarcity is a human construct that can be defined in various ways and water-scarcity footprint results can differ depending upon the particular water-scarcity model chosen. As such, we used three models based on different concepts and with different model structures. The very high correlation between the results obtained using the different models (R = 0.937–0.971) suggests that our results are reliable and not subject to water-scarcity model choice. Another important source of uncertainty is the water use associated with agricultural production and food processing. Steps to quantify and reduce this uncertainty should be a priority for future research.

Our study did not include water use associated with food packaging, as individual foods can be packaged in a very wide variety of ways and information about this subject was not included in the dietary intake survey. Experience suggests that the contribution of retail packaging to the water-scarcity footprint of food products is very minor, often in the order of 1% [24]. Similarly, we did not evaluate food product storage and distribution, kitchen water use associated with food preparation and cleaning of cooking implements and dishes. This may be an important area for water-use efficiency improvement; however, it was deemed outside the scope of the project since this is largely unrelated to dietary strategies. Also, the dietary data collection did not include information about kitchen water use. Finally, our results relate to Australian diets and the Australian food system and results may differ in other countries with different dietary habits and food systems.

## Figures and Tables

**Table 1:** Contribution of different foods (%) to the water scarcity footprint of Australian adult daily diets (*N* = 9341). Food groups are as defined by the Australian Dietary Guidelines.

Food	Male	Female	Total
Fruit	17.5	20.3	18.9
Vegetables	6.5	7.5	7.0
Breads and cereals	13.4	11.8	12.6
Fresh meat and alternatives	12.3	11.0	11.6
Seafood	0.4	0.5	0.5
Beef and lamb	4.1	3.2	3.7
Poultry	3.2	2.8	3.0
Pork	0.7	0.4	0.6
Vegetarian alternatives	3.8	4.0	3.9
Other livestock products	<0.1	<0.1	<0.1
Dairy and alternatives	16.3	15.9	16.1
Beverages	6.2	8.4	7.3
Discretionary foods and beverages	25.9	23.3	24.6
Sugar sweetened beverages	0.9	0.7	0.8
Biscuits, cakes, waffles	2.0	2.4	2.2
Pastries and pies	0.9	0.8	0.9
Processed meat products	6.0	3.9	5.0
Dairy desserts, cream, butter	3.5	2.8	3.2
Fried potato and extruded snacks	2.4	1.6	2.0
Muesli bars, confectionary, chocolate	2.3	2.5	2.4
Alcoholic beverages	6.5	6.8	6.7
Other	1.5	1.7	1.6
Healthy fats and oils	0.8	0.9	0.9
Miscellaneous foods	1.0	0.7	0.9

**Table 2:** Dietary intake (serves person^−1^ day^−1^) and water-scarcity footprint (L-eq person^−1^ day^−1^) of Australian adult (19–50 years) daily diets.

Food Group	Higher Diet Quality/Lower Water-Scarcity Footprint Subgroup (*N* = 683)		Lower Diet Quality/Higher Water-Scarcity Footprint Subgroup (*N* = 538)	
	Serves	Water-Scarcity Footprint	Serves	Water-Scarcity Footprint
Fruit	1.10	35.0	1.76	120.0
Vegetables	3.46	25.0	1.33	15.6
Breads and cereals	3.96	24.2	5.04	90.1
Fresh meat and alternatives	2.66	37.1	1.80	35.9
Dairy and alternatives	1.13	41.6	1.77	70.6
Discretionary food and beverages	2.37	23.9	12.30	207.2
Miscellaneous foods		19.8		34.0
Total		207		573

**Table 3:** Dietary intake (serves person^−1^ day^−1^) and water-scarcity footprint (L-eq person^−1^ day^−1^) for the current and recommended Australian adult (19–50 years) daily diets ^1^.

Food Group	Current Diet (*N* = 5157)		Recommended Diet Average Water-Scarcity Footprint Intensity		Recommended Diet Improved Water-Scarcity Footprint Intensity	
	Serves	Water-Scarcity Footprint	Serves	Water-Scarcity Footprint	Serves	Water-Scarcity Footprint
Fruit	1.38	81.5	2.0	117.9	2.0	64.0
Vegetables	2.47	21.3	5.5	47.3	5.5	39.8
Breads and cereals	4.57	61.6	6.0	80.9	6.0	36.7
Fresh meat and alternatives	2.32	40.7	2.8	49.2	2.8	39.1
Dairy and alternatives	1.46	55.9	2.5	95.6	2.5	92.0
Discretionary food and beverages	7.42	80.6	2.8	30.4	2.8	28.3
Miscellaneous foods		23.7		23.7		19.8
Total		365		445		320

^1^ The improved water-scarcity footprint intensity is based on the higher diet quality/lower water-scarcity footprint subgroup. The number of serves differs marginally for men and women in the recommended Australian diet.

## Data Availability

The dietary intake data are available from the Australian Bureau of Statistics (http://www.abs.gov.au/australianhealthsurvey). Agricultural water-scarcity footprint data are accessible at: https://pubs.acs.org/doi/abs/10.1021/acs.est.8b00416. The water-scarcity footprints of individual foods are presented in the Supplementary Information.

## Supplementary Materials

The following are available online at https://www.mdpi.com/2072-6643/11/8/1846/s1: Table S1: Number of male and female participants; Table S2: Age distribution of participants; Table S3: Number of individuals in the quadrant analysis; Table S4: Water-scarcity footprint of imported commodities; Table S5: Generic aquaculture feed ration; Table S6: Data sources for food processing; Table S7: Specific gravity of common foods; Table S8: Generic recipes used for food products; Table S9: Water-scarcity footprint of individual foods; Table S10: All water-scarcity footprint results; Table S11: Contribution analysis—WSI_HH_EQ; Table S12: Contribution analysis—WORLD_EQ; Table S13: Contribution analysis—AWARE; Table S14: Food intake and water-scarcity footprint—WSI_HH_EQ; Table S15: Food intake and water-scarcity footprint—WORLD_EQ; Table S16: Food intake and water-scarcity footprint—AWARE.
