# Supplementary material for: Diet Quality and Water Scarcity: Evidence from a Large Australian Population Health Survey
Source: Nutrients. 2019 Aug 9;11(8):1846. doi: 10.3390/nu11081846 (PMC6723970; doi:10.3390/nu11081846)
Supplement: Supplementary file 1 [file nutrients-11-01846-s001.pdf]

# Supplementary Information

# Diet quality and water scarcity: Evidence from a large Australian population health survey

Bradley G. Ridoutt[1,2,*], Danielle Baird[3], Kimberley Anastasiou[3] and Gilly A. Hendrie[3]

[1] Commonwealth Scientific and Industrial Research Organisation (CSIRO) Agriculture and Food, Clayton South, Victoria, Australia.

[2] University of the Free State, Department of Agricultural Economics, Bloemfontein, South Africa.

[3] CSIRO Health and Biosecurity, Adelaide, South Australia, Australia.

*Correspondence: brad.ridoutt@csiro.au; Tel.: +61 3 9545 2159

**Contents**


Supplementary Table 1: Number of male and female participants 3

Supplementary Table 2: Age distribution of participants 4

Supplementary Table 3: Number of individuals in the quadrant analysis 5

Supplementary Table 4: Water-scarcity footprint of imported commodities 6

Supplementary Table 5: Generic aquaculture feed ration 7

Supplementary Table 6: Data sources for food processing 8

Supplementary Table 7: Specific gravity of common foods 9

Supplementary Table 8: Generic recipes used for food products 10

Supplementary Table 9: Water-scarcity footprint of individual foods 16

Supplementary Table 10: All water-scarcity footprint results 20

Supplementary Table 11: Contribution analysis – WSI_HH_EQ 21

Supplementary Table 12: Contribution analysis – WORLD_EQ 22

Supplementary Table 13: Contribution analysis – AWARE 23

Supplementary Table 14: Food intake and water-scarcity footprint – WSI_HH_EQ 24

Supplementary Table 15: Food intake and water-scarcity footprint – WORLD_EQ 25

Supplementary Table 16: Food intake and water-scarcity footprint – AWARE 26

Supplementary references 27

**Supplementary Table 1** Number of male and female participants in the study

| | Male | Female | Total |
|---|---|---|---|
| Adults (19 years and older) | 4,282 | 5,059 | 9,341 |
| % | 45.8 | 54.2 | 100 |

**Supplementary Table 2** Age distribution of participants in the study. Age categories are defined according to the Australian dietary guidelines [1].

| Age (years) | Count |
|---|---|
| 19 to 50 | 5,157 |
| 51 to 70 | 2,906 |
| 71+ | 1,278 |
| Total | 9,341 |

**Supplementary Table 3** Number of 19-50 year old adults in the quadrant analysis. Results are presented for 3 water-scarcity models, denoted WSI_HH_EQ [2], World_EQ [3] and AWARE [4]. HDQ and LDQ refer to higher and lower diet quality, respectively. HWF and LWF refer to higher and lower water-scarcity footprint, respectively.

| Water-scarcity model | Quadrant | Count |
|---|---|---|
| WSI_HH_EQ | LDQ-LWF | 1,253 |
| | LDQ-HWF | 585 |
| | HDQ-LWF | 683 |
| | HDQ-HWF | 886 |
| | Within 0.25 SD | 1,750 |
| World_EQ | LDQ-LWF | 1,313 |
| | LDQ-HWF | 538 |
| | HDQ-LWF | 681 |
| | HDQ-HWF | 916 |
| | Within 0.25 SD | 1,709 |
| AWARE | LDQ-LWF | 1,327 |
| | LDQ-HWF | 511 |
| | HDQ-LWF | 715 |
| | HDQ-HWF | 867 |
| | Within 0.25 SD | 1,737 |

**Supplementary Table 4** Water-scarcity footprint (L-eq kg$^{-1}$) of imported agricultural commodities calculated using three water-scarcity models: WSI_HH_EQ [2], World_EQ [3] and AWARE [4]. Agricultural water consumption data [5].

| Commodity | Water-scarcity model | | | Main countries of production |
|---|---|---|---|---|
| | WSI_HH_EQ | World_EQ | AWARE | |
| Tea leaves | 4290 | 3462 | 2414 | China, India, Indonesia, Sri Lanka |
| Coffee (green) | 1078 | 911 | 645 | Brazil, Colombia, India, Indonesia, Papua New Guinea, Vietnam |
| Hops | 20.1 | 91.3 | 78.3 | Czech Republic, Germany, New Zealand |
| Palm oil | 1.4 | 5.3 | 5.2 | Indonesia, Malaysia |
| Cocoa beans | 561 | 335 | 1817 | Brazil, Cameroon, Cote d'Ivoire, Ghana, Indonesia |
| Hazelnut | 1606 | 3143 | 3822 | Turkey, Italy |
| Coconut (desiccated) | 319 | 1275 | 303 | Thailand, Philippines |

Note: Results presented using the AWARE water-scarcity model have been divided by 42.95, the global average value.

**Supplementary Table 5** Generic aquaculture feed ration. Data sources [6-7].

| Component | kg per kg aquaculture production |
|---|---|
| Fish meal | 0.30 |
| Fish oil | 0.13 |
| Plant-based ingredients | 0.36 |
| Winter cereal (50%) | |
| Vegetable oil (20%) | |
| Oilseed meal (30%) | |
| Other animal-based ingredients | 0.52 |
| Chicken meal (100%) | |
| Total | 1.30 |

**Supplementary Table 6** Data sources for water use in food processing.

| Process | Data sources |
|---|---|
| Meat processing | 8-11 |
| Dairy processing | 12 |
| Vegetable processing | 13 |
| Fruit processing | 14-17 |
| Nut processing | 18 |
| Oilseed processing | 18-19 |
| Legume processing | 19 |
| Grain processing | 18-19 |
| Sugar processing | 19 |
| Beverage processing | 20 |
| Food manufacturing<br>Margarine<br>Cereal products<br>Bakery products<br>Processed meat<br>Other foods | 21 |
| Seafood processing | 22 |

**Supplementary Table 7** Specific gravity of common foods and beverages. Source [23].

| Product | Specific gravity |
|---|---|
| Water | 1.000 |
| Carbonated soft drink | 1.041 |
| Sports drink | 1.041 |
| Apple juice | 1.041 |
| Orange juice | 1.041 |
| Other citrus juice | 1.052 |
| Grape juice | 1.052 |
| Pineapple juice | 1.052 |
| Wine | 0.994 |
| Beer | 1.006 |
| Spirits | 0.955 |
| Cider | 1.008 |
| Milk, whole | 1.030 |
| Milk, flavoured | 1.057 |
| Rice beverage | 1.049 |
| Soy beverage | 1.033 |
| Cordial, concentrate, not juice based | 1.672 |
| Cordial, concentrate, juice based | 1.580 |
| Tomato sauce | 1.036 |

**Supplementary Table 8** Generic recipes used for food products

| Food | Ingredient | g $100g^{-1}$ |
|---|---|---|
| Popcorn | Corn | 70 |
| | Vegetable oil | 30 |
| Balsamic vinegar | Fermented grapes | 10 |
| | Water | 90 |
| Juice, fruit & vegetable blend | Vegetable juice | 45 |
| | Apple juice | 25 |
| | Lemon juice | 5 |
| | Orange juice | 25 |
| Sausages | Meat | 75 |
| | Corn flour | 5 |
| | Rice flour | 5 |
| | Water | 15 |
| Liquorice | Flour, wheat | 30 |
| | Sugar | 35 |
| | Water | 20 |
| | Molasses | 15 |
| Rice cake | Rice, brown | 99 |
| | Vegetable oil | 1 |
| Muesli or snack bar, with chocolate chips or coating | Muesli bar with chocolate chips or coating | 80 |
| | Milk chocolate | 20 |
| Soy sauce | Soybeans | 20 |
| | Water | 80 |
| Mayonnaise, regular fat | Sugar | 10 |
| | Egg | 5 |
| | Vegetable oil | 65 |
| | Vinegar | 5 |
| | Water | 15 |
| Extruded snack, cheese flavour | Corn flour | 35 |
| | Rice flour | 35 |
| | Whey powder | 7 |
| | Palm oil | 23 |
| Hazelnut spread | Sugar | 50 |
| | Palm oil | 23 |
| | Hazelnut | 13 |
| | Skim milk powder | 7 |
| | Cocoa powder | 7 |
| Pasty filled with vegetable & meat | Pastry | 40 |
| | Starchy root vegetable | 35 |
| | Beef | 10 |
| | Vegetable oil | 5 |
| | Water | 10 |
| Custard | Milk | 89 |

| | | |
|---|---|---|
| | Sugar | 6 |
| | Thickener, flavours | 5 |
| Danish pastry with custard filling | Custard | 38 |
| | Egg | 8 |
| | Pastry | 48 |
| | Sugar | 6 |
| Apple pie | Apple | 58 |
| | Lemon juice | 1 |
| | Pastry | 35 |
| | Sugar | 5 |
| Quiche Lorraine | Bacon | 13 |
| | Cheese | 9 |
| | Cream | 23 |
| | Egg | 15 |
| | Milk | 10 |
| | Onion | 7 |
| | Pastry | 23 |
| Meat pie | Pastry | 50 |
| | Beef | 25 |
| | Corn flour | 5 |
| | Water | 20 |
| Pastry (shortcrust) | Egg | 2 |
| | Margarine | 9 |
| | Palm oil | 9 |
| | Flour, wheat | 63 |
| | Sugar | 5 |
| | Water | 13 |
| Muffin, cake style with berry, commercial, un-iced | Egg | 14 |
| | Vegetable oil | 17 |
| | Flour, wheat | 20 |
| | Milk | 17 |
| | Berries | 14 |
| | Sugar | 19 |
| Cake or cupcake, chocolate, commercial, sugar-based icing | Cocoa | 5 |
| | Egg | 11 |
| | Flour, wheat | 16 |
| | Milk | 4 |
| | Palm oil | 13 |
| | Sugar | 37 |
| | Water | 13 |
| Cake, lamington, unfilled | Madeira cake | 62 |
| | Coconut | 8 |
| | Sugar | 14 |
| | Cocoa | 5 |
| | Vegetable oil | 1 |

| | | |
|---|---|---|
| | Other | 10 |
| Cake, fruit, commercial, un-iced | Mixed dried fruit | 36 |
| | Flour, wheat | 36 |
| | Sugar | 12 |
| | Vegetable oil | 10 |
| | Egg powder | 6 |
| Biscuit, savoury, corn cake | Maize | 99 |
| | Vegetable oil | 1 |
| Rice cracker, plain | Rice | 197 |
| | Vegetable oil | 511 |
| Biscuit, savoury, white wheat flour, water cracker | Flour, wheat | 88 |
| | Vegetable oil | 12 |
| Cone, for ice-cream | Cornflour | 11 |
| | Wheat flour | 58 |
| | Vegetable oil | 8 |
| | Sugar | 23 |
| Biscuit, sweet, oat | Flour, wheat | 35 |
| | Sugar | 24 |
| | Oats | 13 |
| | Coconut | 8 |
| | Butter | 15 |
| | Vegetable oil | 5 |
| Biscuit, sweet with cream filling | Flour, wheat | 40 |
| | Sugar | 40 |
| | Palm oil | 18 |
| | Cocoa powder | 1 |
| | Condensed milk | 1 |
| Muesli, natural with dried fruit (nut free) | Mixed dried fruit | 20 |
| | Oats | 64 |
| | Wheat bran | 13 |
| | Wheat germ | 3 |
| Pasta, from wheat | Wheat | 100 |
| Chocolate and malt powder | Malt barley | 16 |
| | Rice | 19 |
| | Milk solids | 25 |
| | Sugar | 25 |
| | Cocoa | 15 |
| Madeira cake | Sugar | 27 |
| | Flour, wheat | 34 |
| | Vegetable oil | 14 |
| | Water | 5 |
| | Egg | 20 |
| Croissant | Flour, wheat | 74 |
| | Butter | 18 |
| | Milk powder | 4 |

| | | |
|---|---|---|
| | Sugar | 4 |
| Doughnut, iced | Flour, wheat | 53 |
| | Water | 12 |
| | Vegetable oil | 16 |
| | Sugar | 14 |
| | Other | 5 |
| Biscuit, chocolate coated and cream filled | Flour, wheat | 26 |
| | Milk chocolate | 38 |
| | Sugar | 24 |
| | Vegetable oil | 11 |
| | Other | 1 |
| Biscuit, arrowroot | Condensed milk | 1 |
| | Sugar | 22 |
| | Vegetable oil | 11 |
| | Flour, wheat | 65 |
| | Other | 1 |
| Crispbread, plain | Wheat, flour | 90 |
| | Vegetable oil | 7 |
| | Sugar | 0.4 |
| | Other | 3 |
| Breakfast cereal, mixed grain | Wheat, wholegrain | 47 |
| | Corn | 20 |
| | Mixed dried fruit | 7 |
| | Sugar | 17 |
| | Bran | 7 |
| | Other | 2 |
| Breakfast cereal, corn flakes frosted | Corn | 55 |
| | Sugar | 41 |
| | Other | 4 |
| Breakfast cereal, puffed rice with chocolate | Rice | 59 |
| | Sugar | 37 |
| | Cocoa | 3 |
| | Other | 1 |
| Breakfast cereal, corn flakes | Corn flakes | 89 |
| | Sugar | 8 |
| | Other | 3 |
| Breakfast cereal, puffed rice | Rice | 90 |
| | Sugar | 9 |
| | Other | 1 |
| Breakfast cereal, oat clusters with nuts | Oat flakes | 45 |
| | Barley | 5 |
| | Oat flour | 3 |
| | Sugar | 23 |
| | Vegetable oil | 14 |
| | Rice flour | 5 |

| | | |
|---|---|---|
| Breakfast cereal, oat clusters with fruit | Nuts | 5 |
| | Oat | 58 |
| | Sugar | 21 |
| | Vegetable oil | 10 |
| | Rice flour | 9 |
| | Dried fruit | 2 |
| Breakfast cereal, wheat biscuits | Wheat, wholegrain | 97 |
| | Sugar | 3 |
| Corn chips, without flavouring | Corn | 73 |
| | Oil | 27 |
| Chips (crisps), potato | Potato | 347 |
| | Oil | 38 |
| Sauce, tomato | Tomato | 156 |
| | Sugar | 24 |
| Jam, stone fruit | Sugar | 40 |
| | Fruit | 40 |
| | Water | 20 |
| Cordial, concentrate, citrus base | Sugar | 37 |
| | Orange juice | 13 |
| | Pineapple juice | 13 |
| | Other citrus juice | 6 |
| Cordial, concentrate, not juice based | Sugar | 40 |
| Lolly, jube type | Glucose and dextrose from wheat starch | 25 |
| | Glucose and dextrose from corn starch | 25 |
| | Sugar | 25 |
| Milk chocolate | Milk | 188 |
| | Sugar | 46 |
| | Cocoa products | 30 |
| Tofu | Soy bean | 21 |
| Soy beverage | Soy bean | 4.7 |
| | Vegetable oil | 1 |
| Almond beverage | Almond flour | 3 |
| | Vegetable oil | 0.5 |
| Rice beverage | Rice flour | 14 |
| | Vegetable oil | 0.5 |
| Flavoured milk | Flavour | 1 |
| | Sugar | 5 |
| | Milk | 94 |
| Rum | Molasses | 109 |
| Beer | Hops | 0.25 |
| | Malt (Barley) | 15 |
| Juice (various) | Apple | 154 |
| | Orange | 180 |
| | Lemon | 250 |
| | Grapefruit | 200 |

| | | |
|---|---|---|
| | Citrus, other | 200 |
| | Grape | 133 |
| | Pineapple | 500 |
| | Fruit, other | 200 |
| | Vegetable, mixed | 200 |
| Sports drink | Sugar | 7 |
| Soft drink, sugar sweetened | Sugar | 11 |
| | Canola oil | 50 |
| Margarine | Sunflower oil | 15 |
| | Water | 35 |
| Yogurt, sweetened | Yogurt, plain | 94 |
| | Sugar | 6 |
| Ice-cream, regular | Ice-cream | 87 |
| | Sugar | 13 |

**Supplementary Table 9** Water-scarcity footprint (L-eq kg$^{-1}$) of individual foods calculated using three water-scarcity models: WSI_HH_EQ [2], World_EQ [3] and AWARE [4].

| Food | Description | WSI_HH_EQ | World_EQ | AWARE |
|---|---|---|---|---|
| Almond | Edible portion | 1524.2 | 3447.9 | 10187.6 |
| Almond beverage | | 47.6 | 107.5 | 318.0 |
| Apple juice | | 21.2 | 51.5 | 118.2 |
| Apple pie | | 18.1 | 38.4 | 98.8 |
| Apples | Edible portion | 13.7 | 32.9 | 76.2 |
| Apricot (Dried) | Dried, edible | 1120.3 | 3363.9 | 7758.4 |
| Aquaculture salmon | Edible portion | 15.5 | 51.7 | 89.4 |
| Arrowroot biscuit | | 35.1 | 63.1 | 197.4 |
| Avocado | Edible portion | 113.1 | 120.2 | 287.4 |
| Barley | Uncooked | 6.1 | 22.8 | 43.8 |
| Balsamic vinegar | | 12.5 | 32.2 | 85.8 |
| Beef meat | Fresh, without bone | 39.0 | 131.3 | 258.3 |
| Beef offal | Fresh | 22.1 | 74.6 | 146.7 |
| Beer | | 1.8 | 7.7 | 12.0 |
| Biscuit, chocolate and cream filling | | 145.1 | 216.1 | 662.0 |
| Biscuit, savoury, corn cake | | 40.5 | 95.6 | 282.3 |
| Biscuit, savoury, white wheat flour | | 6.3 | 23.6 | 44.1 |
| Bread, wheat, white | | 3.5 | 13.1 | 24.6 |
| Bread, wheat, wholemeal | | 3.0 | 11.3 | 21.1 |
| Breakfast cereal, puffed rice, chocolate | | 216.9 | 977.1 | 1766.4 |
| Breakfast cereal mix grain | | 71.8 | 188.3 | 468.5 |
| Breakfast cereal, corn flakes | | 37.0 | 83.1 | 244.0 |
| Breakfast cereal, frosted corn flakes | | 39.3 | 63.8 | 199.5 |
| Breakfast cereal, oat cluster fruit | | 83.9 | 277.4 | 602.6 |
| Breakfast cereal, oat cluster nut | | 134.8 | 337.2 | 902.7 |
| Breakfast cereal, puffed rice | | 292.9 | 1464.4 | 2577.6 |
| Breakfast cereal, wheat biscuit | | 5.5 | 16.4 | 33.1 |
| Brown Rice | Uncooked | 321.1 | 1623.4 | 2850.7 |
| Butter | | 241.5 | 1025.6 | 1907.1 |
| Cake or cupcake, chocolate | Sugar based icing | 44.3 | 47.5 | 160.7 |
| Cake, fruit, commercial, un-iced | | 294.3 | 822.1 | 1990.5 |
| Cake, lamington | Unfilled | 70.1 | 156.9 | 195.7 |
| Canola oil | Refined, edible | 36.1 | 136.5 | 255.8 |
| Cheese | | 181.1 | 769.2 | 1430.3 |
| Chips (crisps), potato | | 190.8 | 377.2 | 1070.5 |
| Chocolate and malt powder | Unmixed | 206.2 | 692.9 | 1385.6 |
| Cider | | 21.7 | 54.2 | 120.9 |
| Citrus fruit | Edible portion | 116.3 | 222.3 | 743.0 |
| Cocoa powder | Unsweetened | 363.3 | 216.6 | 1175.9 |
| Coconut, desiccated | | 319.3 | 1275.2 | 302.7 |
| Coffee | Roasted bean | 1283.1 | 1085.1 | 767.3 |
| Cone for ice-cream | Cone only | 28.4 | 47.6 | 150.6 |

| | | | | |
|---|---|---|---|---|
| Cordial, 50% juice, citrus | Concentrate, unmixed | 72.7 | 111.1 | 361.1 |
| Cordial, lime flavouring | Concentrate, unmixed | 18.4 | 14.7 | 55.0 |
| Corn flour | | 37.3 | 89.9 | 261.6 |
| Corn chips | | 78.9 | 166.3 | 534.6 |
| Cream | | 135.0 | 573.1 | 1065.7 |
| Crispbread, wheat based, refined | | 17.3 | 41.6 | 116.4 |
| Crispbread, wholegrain | | 23.4 | 49.4 | 153.9 |
| Croissant | | 60.0 | 246.9 | 462.2 |
| Custard | | 34.3 | 136.3 | 257.7 |
| Danish pastry with custard | | 27.3 | 79.8 | 169.9 |
| Eggs | Whole, without shell | 62.8 | 127.8 | 313.7 |
| Extruded cheese snack | | 160.7 | 762.3 | 1382.3 |
| Flavoured milk, chocolate | | 39.3 | 145.7 | 282.2 |
| Flavoured milk, other | | 35.6 | 143.6 | 270.5 |
| Game meat | | 1.2 | 7.6 | 5.9 |
| Grape juice | | 122.5 | 304.7 | 847.4 |
| Grapes | Edible portion | 92.0 | 228.5 | 636.5 |
| Hazelnut spread | | 555.4 | 1066.1 | 1459.7 |
| Honey | | 0.2 | 1.0 | 0.6 |
| Ice-cream regular fat | | 61.5 | 240.1 | 455.9 |
| Iced doughnut | | 39.1 | 73.3 | 237.2 |
| Jam, stone fruit | | 104.5 | 273.3 | 651.1 |
| Juice, fruit & vegetable blend | | 109.5 | 215.2 | 638.0 |
| Lamb meat | Fresh, without bone | 27.7 | 60.6 | 128.5 |
| Lamb offal | Fresh | 12.6 | 27.5 | 58.4 |
| Liquorice | | 18.8 | 18.7 | 61.8 |
| Lobster | Edible portion | 2.8 | 13.4 | 9.3 |
| Lolly, jube type | | 24.9 | 44.9 | 127.8 |
| Madeira cake | | 31.6 | 60.4 | 146.9 |
| Margarine | | 70.4 | 160.8 | 473.1 |
| Mayonnaise | Regular fat | 233.3 | 407.8 | 1521.5 |
| Meat pie | | 17.8 | 52.2 | 114.3 |
| Milk chocolate | | 292.5 | 421.8 | 1251.3 |
| Muesli or snack bar, with chocolate chips or coating | | 158.5 | 312.4 | 918.6 |
| Muesli, natural with dried fruit (nut free) | | 160.5 | 463.4 | 1112.3 |
| Muffin, cake-style, berry, commercial, un-iced | | 85.7 | 203.9 | 539.7 |
| Muesli bar with fruit/nut | | 271.7 | 646.6 | 1827.8 |
| Muesli bar with nuts/seeds | | 125.0 | 284.9 | 835.4 |
| Oat biscuit, sweet | | 76.0 | 255.1 | 355.1 |
| Oats | Uncooked | 6.3 | 23.4 | 45.1 |
| Olive oil | | 962.8 | 2890.7 | 6668.0 |
| Orange juice | | 209.5 | 401.0 | 1338.3 |
| Other citrus juice | | 232.7 | 445.4 | 1486.9 |
| Other nut | Edible portion | 1020.7 | 2308.9 | 6822.2 |
| Palm oil | | 1.4 | 5.3 | 5.2 |
| Pasta from wheat | Dry, uncooked | 4.9 | 18.4 | 35.0 |
| Pastry (shortcrust) | | 13.1 | 31.1 | 78.3 |
| Pasty, filled with vegetables & meat | | 32.7 | 72.4 | 190.6 |

| | | | | |
|---|---|---|---|---|
| Peanut | Edible portion | 186.7 | 744.3 | 1398.2 |
| Peanut oil | Refined, edible | 345.6 | 1377.9 | 2588.3 |
| Pears | Edible portion | 9.6 | 32.0 | 61.7 |
| Pig meat | Fresh, without bone | 44.8 | 94.7 | 235.9 |
| Pig offal | Fresh | 8.6 | 18.2 | 45.4 |
| Pineapple juice | | 115.5 | 146.9 | 373.1 |
| Plantation fruit | Edible portion | 23.1 | 29.2 | 74.4 |
| Popcorn | | 64.7 | 138.3 | 438.9 |
| Poultry meat | Fresh, without bone | 41.4 | 95.2 | 216.0 |
| Poultry offal | Fresh | 21.1 | 48.4 | 109.7 |
| Prawn | Edible portion | 3.1 | 14.7 | 10.2 |
| Processed beef products | | 40.6 | 141.6 | 266.3 |
| Processed lamb products | | 29.3 | 71.0 | 136.5 |
| Processed pig meat products | | 46.4 | 105.1 | 243.9 |
| Processed poultry products | | 43.1 | 105.5 | 223.9 |
| Quiche Lorraine | | 72.6 | 263.0 | 515.0 |
| Reduced fat milk | | 24.9 | 105.6 | 196.3 |
| Rice cake, wholegrain | | 319.8 | 1611.4 | 2835.3 |
| Rice cracker, plain | | 366.4 | 1818.5 | 3229.4 |
| Rice flour | | 366.9 | 1855.4 | 3258.0 |
| Rice milk | | 46.9 | 231.4 | 411.5 |
| Rum | | 12.5 | 20.7 | 43.3 |
| Rye flour | | 5.5 | 20.6 | 39.7 |
| Safflower oil | Refined, edible | 38.7 | 146.1 | 273.8 |
| Sauce, tomato, commercial | | 42.0 | 97.5 | 230.7 |
| Sausage, beef | | 51.1 | 206.1 | 377.7 |
| Sausage, chicken | | 52.9 | 179.0 | 345.9 |
| Sausage, lamb | | 42.6 | 153.1 | 280.3 |
| Sausage, pork | | 55.4 | 178.7 | 360.9 |
| Soft drink, diet | | 0.2 | 1.2 | 1.3 |
| Soft drink, sugar sweetened | | 5.2 | 5.0 | 16.0 |
| Soy milk | | 10.1 | 33.2 | 72.6 |
| Soy sauce | | 25.2 | 100.8 | 188.1 |
| Soybean oil | Refined, edible | 268.1 | 1068.9 | 2007.8 |
| Sports drink | | 3.4 | 3.6 | 10.6 |
| Starchy root veg | Edible portion | 39.9 | 79.6 | 198.4 |
| Stone fruit | Edible portion, includes berries | 215.4 | 646.9 | 1492.0 |
| Sugar | Refined | 45.4 | 33.9 | 134.1 |
| Sultana (Dried grape) | Dried, edible | 432.2 | 1073.8 | 2991.6 |
| Sunflower oil | Refined, edible | 347.7 | 611.5 | 2297.0 |
| Sweet biscuit with cream filling | | 24.8 | 26.6 | 85.1 |
| Tea | Dried leaves | 4289.9 | 3462.1 | 2414.4 |
| Tofu | | 29.4 | 117.7 | 219.9 |
| Tropical stone fruit | Edible portion | 119.5 | 125.3 | 302.9 |
| Veg, excluding starchy roots and avocado | Edible portion | 44.5 | 88.2 | 221.2 |
| Water, bottled | | 0.2 | 0.9 | 0.9 |
| Water, tap | | 0.1 | 0.6 | 0.6 |
| Wheat bran | | 6.3 | 23.5 | 45.3 |

| | | | | |
|---|---|---|---|---|
| Wheat flour, white | | 4.9 | 18.1 | 34.8 |
| Wheat flour, wholegrain | | 4.2 | 15.5 | 29.8 |
| White rice | Uncooked | 366.9 | 1855.4 | 3258.0 |
| Whole milk | | 35.5 | 150.8 | 280.5 |
| Whole milk powder | | 277.0 | 1176.4 | 2187.6 |
| Wild capture fish | Edible portion | 5.2 | 24.4 | 16.9 |
| Wine | Red or white | 131.7 | 328.3 | 911.3 |
| Winter legumes | Lupin, chickpea, faba, Lentil (canned/soaked) | 2.6 | 5.9 | 11.3 |
| Yogurt, plain | | 39.1 | 165.9 | 308.5 |
| Yogurt, sweetened | | 39.4 | 158.0 | 298.0 |

Note: Results presented using the AWARE water-scarcity model have been divided by 42.95, the global average value.

**Supplementary Table 10** Water-scarcity footprint results for Australian adult daily diets. Results are presented for 3 water-scarcity models, denoted WSI_HH_EQ [2], World_EQ [3] and AWARE [4]. Age categories are defined according to the Australian dietary guidelines [1].

| Age | Water-scarcity model | Male | | Female | | Total | |
|---|---|---|---|---|---|---|---|
| | | Mean | SD | Mean | SD | Mean | SD |
| 19 to 50 | WSI_HH_EQ | 143.3 | 80.5 | 134.8 | 74.8 | 139.2 | 77.9 |
| | World_EQ | 386.3 | 232.4 | 343.3 | 209.9 | 365.3 | 222.7 |
| | AWARE | 837.4 | 523.0 | 753.3 | 483.5 | 796.4 | 505.9 |
| 51 to 70 | WSI_HH_EQ | 154.1 | 83.5 | 154.2 | 80.1 | 154.1 | 81.8 |
| | World_EQ | 379.9 | 227.4 | 365.8 | 213.7 | 372.8 | 220.8 |
| | AWARE | 835.2 | 537.2 | 807.8 | 511.1 | 821.5 | 524.5 |
| 71+ | WSI_HH_EQ | 135.0 | 67.2 | 131.7 | 68.2 | 133.1 | 67.7 |
| | World_EQ | 323.9 | 175.1 | 309.7 | 179.8 | 315.8 | 177.9 |
| | AWARE | 713.2 | 426.8 | 673.9 | 438.7 | 690.9 | 434.0 |
| All adults | WSI_HH_EQ | 145.6 | 80.5 | 139.8 | 76.1 | 142.7 | 78.4 |
| | World_EQ | 379.1 | 227.3 | 345.6 | 208.4 | 362.4 | 218.7 |
| | AWARE | 826.1 | 520.5 | 759.3 | 488.0 | 792.8 | 505.7 |

Note: Results presented using the AWARE water-scarcity model have been divided by 42.95, the global average value

**Supplementary Table 11:** Contribution of different foods (%) to the water-scarcity footprint calculated using the WSI_HH_EQ [2] model.

| | 19-50 years | | | 51-70 years | | | 71 years and older | | | Total | | |
|---|---|---|---|---|---|---|---|---|---|---|---|---|
| | Male | Female | Total | Male | Female | Total | Male | Female | Total | Male | Female | Total |
| Fruit | 16.8 | 19.1 | 17.9 | 16.7 | 20.0 | 18.3 | 22.2 | 21.2 | 21.6 | 17.2 | 19.6 | 18.4 |
| Vegetables | 7.0 | 8.1 | 7.5 | 7.8 | 7.9 | 7.8 | 9.1 | 8.7 | 8.9 | 7.4 | 8.1 | 7.8 |
| Breads and cereals | 11.4 | 9.7 | 10.6 | 7.6 | 6.4 | 7.0 | 5.4 | 5.2 | 5.3 | 9.8 | 8.3 | 9.1 |
| Meat and alternatives | 12.2 | 10.3 | 11.3 | 12.1 | 10.8 | 11.4 | 9.1 | 9.2 | 9.1 | 11.9 | 10.3 | 11.1 |
| Fish | 0.3 | 0.3 | 0.3 | 0.4 | 0.5 | 0.5 | 0.2 | 0.3 | 0.2 | 0.3 | 0.4 | 0.4 |
| Red meat (no pork) | 3.5 | 2.6 | 3.1 | 3.8 | 2.9 | 3.3 | 3.1 | 2.6 | 2.8 | 3.6 | 2.7 | 3.1 |
| Poultry | 4.0 | 3.1 | 3.5 | 2.4 | 2.4 | 2.4 | 2.3 | 2.1 | 2.1 | 3.4 | 2.8 | 3.1 |
| Vegetarian meat alt and nuts | 3.6 | 3.9 | 3.7 | 4.9 | 4.5 | 4.7 | 3.3 | 3.7 | 3.5 | 3.9 | 4.1 | 4.0 |
| Other | 0.0 | 0.0 | 0.0 | 0.0 | 0.0 | 0.0 | 0.0 | 0.0 | 0.0 | 0.0 | 0.0 | 0.0 |
| Pork | 0.9 | 0.4 | 0.6 | 0.6 | 0.5 | 0.5 | 0.3 | 0.5 | 0.4 | 0.7 | 0.4 | 0.6 |
| Dairy | 13.6 | 11.8 | 12.7 | 9.1 | 9.9 | 9.5 | 9.2 | 9.7 | 9.4 | 12.0 | 11.0 | 11.5 |
| Beverages | 10.8 | 15.6 | 13.1 | 20.4 | 22.3 | 21.4 | 21.4 | 26.2 | 24.1 | 14.3 | 18.7 | 16.5 |
| Discretionary foods | 26.4 | 23.9 | 25.2 | 24.8 | 20.8 | 22.8 | 22.0 | 18.4 | 20.0 | 25.6 | 22.4 | 24.0 |
| Sugar sweetened beverages | 2.2 | 1.5 | 1.8 | 1.1 | 0.7 | 0.9 | 0.7 | 0.6 | 0.6 | 1.7 | 1.2 | 1.5 |
| Savoury and sweet biscuits | 2.0 | 2.4 | 2.2 | 2.0 | 2.0 | 2.0 | 2.8 | 2.9 | 2.8 | 2.1 | 2.3 | 2.2 |
| Sweet and savoury pastries and pies | 0.9 | 0.8 | 0.8 | 0.9 | 0.6 | 0.8 | 0.6 | 0.4 | 0.5 | 0.9 | 0.7 | 0.8 |
| Processed meat, burgers, tacos, pizza | 6.2 | 4.1 | 5.2 | 3.3 | 1.8 | 2.6 | 2.5 | 2.1 | 2.3 | 5.1 | 3.3 | 4.2 |
| Dairy based desserts, cream, butter | 2.0 | 1.8 | 1.9 | 3.0 | 1.7 | 2.3 | 3.5 | 2.7 | 3.1 | 2.4 | 1.9 | 2.1 |
| Fried potato and extruded snacks | 3.6 | 2.3 | 3.0 | 1.7 | 0.7 | 1.2 | 0.8 | 0.7 | 0.8 | 2.8 | 1.7 | 2.3 |
| Muesli bars, confectionary and choc | 3.0 | 3.4 | 3.2 | 2.9 | 2.9 | 2.9 | 2.4 | 2.1 | 2.2 | 3.0 | 3.1 | 3.0 |
| Alcohol | 4.8 | 5.8 | 5.3 | 8.6 | 8.8 | 8.7 | 7.2 | 5.2 | 6.1 | 6.0 | 6.5 | 6.3 |
| Other - stock cubes, salt, dry soups, etc. | 1.7 | 1.8 | 1.8 | 1.3 | 1.6 | 1.4 | 1.6 | 1.8 | 1.7 | 1.6 | 1.8 | 1.7 |
| Healthy fats and oils | 0.8 | 1.0 | 0.9 | 1.2 | 1.3 | 1.2 | 1.1 | 1.0 | 1.0 | 0.9 | 1.1 | 1.0 |
| Misc. | 1.0 | 0.6 | 0.8 | 0.4 | 0.6 | 0.5 | 0.7 | 0.3 | 0.5 | 0.8 | 0.5 | 0.7 |

**Supplementary Table 12:** Contribution of different foods (%) to the water-scarcity footprint calculated using the World_EQ [3] model.

| | 19-50 years | | | 51-70 years | | | 71 years and older | | | Total | | |
|---|---|---|---|---|---|---|---|---|---|---|---|---|
| | Male | Female | Total | Male | Female | Total | Male | Female | Total | Male | Female | Total |
| Fruit | 16.6 | 19.3 | 17.9 | 17.7 | 21.7 | 19.7 | 23.6 | 22.9 | 23.2 | 17.5 | 20.3 | 18.9 |
| Vegetables | 6.1 | 7.4 | 6.7 | 7.1 | 7.4 | 7.3 | 8.4 | 8.3 | 8.3 | 6.5 | 7.5 | 7.0 |
| Breads and cereals | 15.1 | 13.7 | 14.4 | 11.1 | 9.3 | 10.2 | 7.7 | 7.8 | 7.8 | 13.4 | 11.8 | 12.6 |
| Meat and alternatives | 12.2 | 10.7 | 11.5 | 13.1 | 12.1 | 12.6 | 9.9 | 10.2 | 10.1 | 12.3 | 11.0 | 11.6 |
| Fish | 0.4 | 0.4 | 0.4 | 0.6 | 0.7 | 0.6 | 0.3 | 0.4 | 0.4 | 0.4 | 0.5 | 0.5 |
| Red meat (no pork) | 3.9 | 3.0 | 3.5 | 4.7 | 3.7 | 4.2 | 3.7 | 3.3 | 3.5 | 4.1 | 3.2 | 3.7 |
| Poultry | 3.6 | 3.1 | 3.4 | 2.4 | 2.6 | 2.5 | 2.4 | 2.3 | 2.3 | 3.2 | 2.8 | 3.0 |
| Vegetarian meat alt and nuts | 3.4 | 3.8 | 3.6 | 5.0 | 4.6 | 4.8 | 3.3 | 3.7 | 3.5 | 3.8 | 4.0 | 3.9 |
| Other | 0.0 | 0.0 | 0.0 | 0.0 | 0.0 | 0.0 | 0.0 | 0.0 | 0.0 | 0.0 | 0.0 | 0.0 |
| Pork | 0.8 | 0.4 | 0.6 | 0.5 | 0.5 | 0.5 | 0.3 | 0.5 | 0.4 | 0.7 | 0.4 | 0.6 |
| Dairy | 17.6 | 16.3 | 17.0 | 13.8 | 15.1 | 14.4 | 15.0 | 15.9 | 15.5 | 16.3 | 15.9 | 16.1 |
| Beverages | 4.8 | 7.0 | 5.9 | 8.8 | 9.9 | 9.4 | 8.8 | 12.2 | 10.7 | 6.2 | 8.4 | 7.3 |
| Discretionary foods | 25.7 | 24.1 | 24.9 | 26.7 | 22.4 | 24.6 | 24.8 | 21.2 | 22.8 | 25.9 | 23.3 | 24.6 |
| Sugar sweetened beverages | 1.1 | 0.8 | 1.0 | 0.6 | 0.4 | 0.5 | 0.4 | 0.4 | 0.4 | 0.9 | 0.7 | 0.8 |
| Savoury and sweet biscuits | 1.8 | 2.4 | 2.1 | 2.1 | 2.2 | 2.1 | 2.8 | 3.1 | 3.0 | 2.0 | 2.4 | 2.2 |
| Sweet and savoury pastries and pies | 0.9 | 0.9 | 0.9 | 1.0 | 0.7 | 0.8 | 0.7 | 0.5 | 0.6 | 0.9 | 0.8 | 0.9 |
| Processed meat, burgers, tacos, pizza | 7.1 | 4.8 | 6.0 | 4.3 | 2.4 | 3.3 | 3.4 | 2.9 | 3.1 | 6.0 | 3.9 | 5.0 |
| Dairy based desserts, cream, butter | 2.8 | 2.6 | 2.7 | 4.4 | 2.8 | 3.6 | 5.6 | 4.5 | 5.0 | 3.5 | 2.8 | 3.2 |
| Fried potato and extruded snacks | 3.0 | 2.1 | 2.6 | 1.4 | 0.7 | 1.1 | 0.8 | 0.8 | 0.8 | 2.4 | 1.6 | 2.0 |
| Muesli bars, confectionary and choc | 2.3 | 2.7 | 2.5 | 2.3 | 2.4 | 2.3 | 1.9 | 1.8 | 1.8 | 2.3 | 2.5 | 2.4 |
| Alcohol | 5.1 | 5.9 | 5.5 | 9.5 | 9.3 | 9.4 | 7.8 | 5.6 | 6.5 | 6.5 | 6.8 | 6.7 |
| Other - stock cubes, salt, dry soups, etc. | 1.6 | 1.8 | 1.7 | 1.3 | 1.6 | 1.4 | 1.5 | 1.7 | 1.6 | 1.5 | 1.7 | 1.6 |
| Healthy fats and oils | 0.7 | 0.8 | 0.8 | 1.1 | 1.2 | 1.2 | 1.0 | 0.9 | 1.0 | 0.8 | 0.9 | 0.9 |
| Misc. | 1.3 | 0.7 | 1.0 | 0.4 | 0.8 | 0.6 | 0.7 | 0.5 | 0.6 | 1.0 | 0.7 | 0.9 |

**Supplementary Table 13:** Contribution of different foods (%) to the water-scarcity footprint calculated using the AWARE [4] model.

| | 19-50 years | | | 51-70 years | | | 71 years and older | | | Total | | |
|---|---|---|---|---|---|---|---|---|---|---|---|---|
| | Male | Female | Total | Male | Female | Total | Male | Female | Total | Male | Female | Total |
| Fruit | 18.2 | 21.0 | 19.6 | 19.5 | 23.7 | 21.6 | 26.0 | 25.6 | 25.8 | 19.2 | 22.3 | 20.7 |
| Vegetables | 6.9 | 8.3 | 7.6 | 8.2 | 8.6 | 8.4 | 9.7 | 9.8 | 9.7 | 7.5 | 8.5 | 8.0 |
| Breads and cereals | 14.3 | 12.9 | 13.6 | 10.6 | 8.9 | 9.8 | 7.5 | 7.5 | 7.5 | 12.7 | 11.2 | 12.0 |
| Meat and alternatives | 12.9 | 11.6 | 12.3 | 14.3 | 13.3 | 13.8 | 10.6 | 11.5 | 11.1 | 13.1 | 12.0 | 12.6 |
| Fish | 0.3 | 0.3 | 0.3 | 0.4 | 0.6 | 0.5 | 0.2 | 0.3 | 0.2 | 0.3 | 0.4 | 0.4 |
| Red meat (no pork) | 3.9 | 3.0 | 3.5 | 4.8 | 3.7 | 4.3 | 3.8 | 3.5 | 3.6 | 4.1 | 3.3 | 3.7 |
| Poultry | 3.9 | 3.3 | 3.6 | 2.6 | 2.9 | 2.7 | 2.5 | 2.6 | 2.5 | 3.4 | 3.1 | 3.3 |
| Vegetarian meat alt and nuts | 3.9 | 4.5 | 4.2 | 5.8 | 5.6 | 5.7 | 3.8 | 4.5 | 4.2 | 4.4 | 4.8 | 4.6 |
| Other | 0.0 | 0.0 | 0.0 | 0.0 | 0.0 | 0.0 | 0.0 | 0.0 | 0.0 | 0.0 | 0.0 | 0.0 |
| Pork | 0.9 | 0.4 | 0.6 | 0.6 | 0.5 | 0.6 | 0.3 | 0.5 | 0.5 | 0.8 | 0.5 | 0.6 |
| Dairy | 15.5 | 14.4 | 14.9 | 12.3 | 13.6 | 13.0 | 13.5 | 14.5 | 14.1 | 14.4 | 14.2 | 14.3 |
| Beverages | 2.1 | 3.0 | 2.6 | 3.7 | 4.1 | 3.9 | 3.5 | 5.5 | 4.7 | 2.7 | 3.6 | 3.1 |
| Discretionary foods | 28.2 | 27.1 | 27.6 | 29.7 | 25.6 | 27.6 | 27.2 | 24.0 | 25.4 | 28.5 | 26.3 | 27.4 |
| Sugar sweetened beverages | 1.4 | 1.1 | 1.3 | 0.8 | 0.5 | 0.7 | 0.5 | 0.5 | 0.5 | 1.2 | 0.9 | 1.0 |
| Savoury and sweet biscuits | 2.1 | 2.7 | 2.4 | 2.3 | 2.5 | 2.4 | 3.3 | 3.6 | 3.5 | 2.3 | 2.8 | 2.5 |
| Sweet and savoury pastries and pies | 1.1 | 1.0 | 1.0 | 1.1 | 0.8 | 1.0 | 0.7 | 0.5 | 0.6 | 1.1 | 0.9 | 1.0 |
| Processed meat, burgers, tacos, pizza | 7.0 | 4.8 | 5.9 | 4.3 | 2.4 | 3.4 | 3.3 | 2.9 | 3.1 | 6.0 | 3.9 | 5.0 |
| Dairy based desserts, cream, butter | 2.6 | 2.4 | 2.5 | 4.2 | 2.6 | 3.4 | 5.3 | 4.3 | 4.7 | 3.2 | 2.6 | 2.9 |
| Fried potato and extruded snacks | 3.9 | 2.7 | 3.3 | 2.0 | 0.9 | 1.4 | 1.1 | 1.0 | 1.0 | 3.1 | 2.0 | 2.6 |
| Muesli bars, confectionary and choc | 2.9 | 3.5 | 3.2 | 3.0 | 3.0 | 3.0 | 2.3 | 2.3 | 2.3 | 2.8 | 3.2 | 3.0 |
| Alcohol | 5.3 | 6.7 | 6.0 | 10.4 | 10.7 | 10.5 | 8.8 | 6.5 | 7.5 | 7.0 | 7.8 | 7.4 |
| Other - stock cubes, salt, dry soups, etc. | 1.9 | 2.2 | 2.1 | 1.6 | 2.1 | 1.9 | 1.7 | 2.5 | 2.2 | 1.8 | 2.2 | 2.0 |
| Healthy fats and oils | 0.8 | 1.0 | 0.9 | 1.3 | 1.4 | 1.4 | 1.2 | 1.1 | 1.2 | 1.0 | 1.1 | 1.1 |
| Misc. | 1.2 | 0.7 | 0.9 | 0.4 | 0.8 | 0.6 | 0.7 | 0.5 | 0.6 | 0.9 | 0.7 | 0.8 |

Note: Results presented using the AWARE water-scarcity model have been divided by 42.95, the global average value.

**Supplementary Table 14:** Food intake (serves person$^{-1}$) and water-scarcity footprint (L-eq person$^{-1}$) for Australian adult (19-50 years) daily diets. Four dietary patterns are compared: higher diet quality and lower water-scarcity footprint, lower diet quality and higher water-scarcity footprint, current diet and recommended diet as described by the Australian dietary guidelines[1]. Water-scarcity footprints were calculated using the WSI_HH_EQ model [2].

| | Higher diet quality & lower water-scarcity footprint | | Lower diet quality & higher water-scarcity footprint | | Current diet | | Recommended diet | |
|---|---|---|---|---|---|---|---|---|
| | Serves | Water-scarcity footprint | Serves | Water-scarcity footprint | Serves | Water-scarcity footprint | Serves | Water-scarcity footprint |
| Fruit | 1.06 | 14.2 | 1.79 | 49.6 | 1.38 | 31.1 | 2 | 45.0 |
| Vegetables | 3.07 | 10.5 | 1.47 | 7.6 | 2.47 | 9.7 | 5.5 | 21.5 |
| Breads and cereals | 4.38 | 9.6 | 4.51 | 17.4 | 4.57 | 14.8 | 6 | 19.4 |
| Fresh meat & alternatives | 2.62 | 13.2 | 1.82 | 13.4 | 2.32 | 16.0 | 2.8 | 19.3 |
| Dairy products & alternatives | 1.26 | 12.6 | 1.59 | 19.1 | 1.46 | 16.2 | 2.5 | 27.7 |
| Discretionary foods | 2.25 | 9.3 | 12.52 | 80.6 | 7.42 | 32.3 | 2.8 | 12.2 |
| Miscellaneous foods | | 13.9 | | 28.6 | | 19.1 | | 19.1 |
| TOTAL | | 83.3 | | 216.3 | | 139.2 | | 164.3 |

**Supplementary Table 15:** Food intake (serves $person^{-1}$) and water-scarcity footprint (L-eq $person^{-1}$) for Australian adult (19-50 years) daily diets. Four dietary patterns are compared: higher diet quality and lower water-scarcity footprint, lower diet quality and higher water-scarcity footprint, current diet and recommended diet as described by the Australian dietary guidelines[1]. Water-scarcity footprints were calculated using the World_EQ model [3].

| | Higher diet quality & lower water-scarcity footprint | | Lower diet quality & higher water-scarcity footprint | | Current diet | | Recommended diet | |
|---|---|---|---|---|---|---|---|---|
| | Serves | Water-scarcity footprint | Serves | Water-scarcity footprint | Serves | Water-scarcity footprint | Serves | Water-scarcity footprint |
| Fruit | 1.10 | 35.0 | 1.76 | 120.0 | 1.38 | 81.5 | 2.0 | 117.9 |
| Vegetables | 3.46 | 25.0 | 1.33 | 15.6 | 2.47 | 21.3 | 5.5 | 47.3 |
| Breads and cereals | 3.96 | 24.2 | 5.04 | 90.1 | 4.57 | 61.6 | 6.0 | 80.9 |
| Fresh meat & alternatives | 2.66 | 37.1 | 1.80 | 35.9 | 2.32 | 40.7 | 2.8 | 49.2 |
| Dairy products & alternatives | 1.13 | 41.6 | 1.77 | 70.6 | 1.46 | 55.9 | 2.5 | 95.6 |
| Discretionary foods | 2.37 | 23.9 | 12.30 | 207.2 | 7.42 | 80.6 | 2.8 | 30.4 |
| Miscellaneous foods | | 19.8 | | 34.0 | | 23.7 | | 23.7 |
| TOTAL | | 206.7 | | 573.4 | | 365.3 | | 445.1 |

**Supplementary Table 16:** Food intake (serves $person^{-1}$) and water-scarcity footprint (L-eq $person^{-1}$) for Australian adult (19-50 years) daily diets. Four dietary patterns are compared: higher diet quality and lower water-scarcity footprint, lower diet quality and higher water-scarcity footprint, current diet and recommended diet as described by the Australian dietary guidelines[1]. Water-scarcity footprints were calculated using the AWARE [4] model.

| | Higher diet quality & lower water-scarcity footprint | | Lower diet quality & higher water-scarcity footprint | | Current diet | | Recommended diet | |
|---|---|---|---|---|---|---|---|---|
| | Serves | Water-scarcity footprint | Serves | Water-scarcity footprint | Serves | Water-scarcity footprint | Serves | Water-scarcity footprint |
| Fruit | 1.08 | 83.4 | 2.00 | 352.0 | 1.38 | 199.9 | 2 | 289.3 |
| Vegetables | 3.40 | 59.7 | 1.32 | 37.2 | 2.47 | 51.3 | 5.5 | 114.1 |
| Breads and cereals | 4.14 | 58.6 | 4.68 | 142.5 | 4.57 | 116.4 | 6 | 153.0 |
| Fresh meat & alternatives | 2.67 | 79.4 | 1.65 | 72.0 | 2.32 | 97.7 | 2.8 | 118.0 |
| Dairy products & alternatives | 1.19 | 79.3 | 1.59 | 115.9 | 1.46 | 102.0 | 2.5 | 174.4 |
| Discretionary foods | 2.28 | 55.5 | 12.94 | 545.5 | 7.42 | 199.3 | 2.8 | 75.2 |
| Miscellaneous foods | | 25.4 | | 41.7 | | 29.8 | | 29.8 |
| TOTAL | | 441.2 | | 1306.8 | | 796.4 | | 953.8 |

Note: Results presented using the AWARE water-scarcity model have been divided by 42.95, the global average value

**Supplementary references**


1. *Australian Dietary Guidelines Summary*, National Health and Medical Research Council: Canberra, 2013.
2. Ridoutt, B.G.; Pfister, S. Water footprinting using a water stress index (WSI) that integrates stress on humans and ecosystems. Proceedings of the 3rd LCA AgriFood Asia Conference, Bangkok, Thailand, 2014.
3. Ridoutt, B.G.; Pfister, S. A revised approach to water footprinting to make transparent the impacts of consumption and production on global freshwater scarcity. *Glob. Environ. Change* **2010**, *20*, 113–20.
4. Boulay, A.M.; Bare, J.; Benini, L.; Berger, M.; Lathuillière, M.J.; Manzardo, A.; Margni, M.; Motoshita, M.; Núñez, M.; Pastor, A.V.; Ridoutt, B.; Oki, T.; Worbe, S.; Pfister, S. The WULCA consensus characterization model for water scarcity footprints: assessing impacts of water consumption based on available water remaining (AWARE). *Int. J. Life Cycle. Assess* **2018***, 23*, 368-378.
5. Pfister, S.; Bayer, P.; Koehler, A.; Hellweg, S. Environmental impacts of water use in global crop production: Hotspots and trade-offs with land use. *Environ. Sci. Technol.* **2011**, *45*, 5761-5768.
6. *TASSAL Sustainability Report 2011*, Tassal Group Limited: Australia, 2011.
7. Pahlow, M.; van Oel, P.R.; Mekonnen, M.M.; Hoekstra, A.Y. Increasing pressure on freshwater resources due to terrestrial feed ingredients for aquaculture production. *Sci. Tot. Environ.* **2015**, *536*, 847-857.
8. Ridoutt, B.; Sanguansri, P.; Alexander, D. *Environmental Performance Review: Red Meat Processing Sector 2015,* Australian Meat Processing Corporation: North Sydney, Australia, 2015.

9. Wiedmann, S.; Yan, M. Livestock meat processing: inventory data and methods for handling co-production for major livestock species and meat products. Proceedings of the 9th International Conference on Life Cycle Assessment in the Agri-Food Sector, San Francisco, USA, 2014.
10. Wiedemann, S.; McGahan, E.; Poad, G. *Using Life Cycle Assessment to Quantify the Environmental Impact of Chicken Meat Production*, Australian Government, Rural Industries Research and Development Corporation: Canberra, 2012.
11. Wiedemann, S.; McGahan, E.; Grist, S.; Grant, T. *Environmental Assessment of Two Pork Supply Chains Using Life Cycle Assessment*, Australian Government, Rural Industries Research and Development Corporation: Canberra, 2010.
12. *Environmental Sustainability Scorecard 2016-17,* Dairy Manufacturers Sustainability Council: Melbourne, Australia, 2018.
13. Lehto, M.; Sipilä, I.; Alakukku, L.; Kymäläinen, H.-R. Water consumption and wastewaters in fresh-cut vegetable production. *Agric. Food Sci.* **2014**, *23*, 246-256.
14. Vinyes, E.; Asin, L.; Alegre, S.; Muños, P.; Boschmonart, J. Life cycle assessment of apple and peach production, distribution and consumption in Mediterranean fruit sector. *J. Clean. Prod.* **2017***, 149*, 313-320.
15. Walker, C.; Beretta, C.; Sanjuán, N.; Hellweg, S. Calculating the energy and water use in food processing and assessing the resulting impacts. *Int. J. Life Cycle Assess.* **2018***, 23*, 824-839.
16. Ingwersen, W.W. Life cycle assessment of fresh pineapple from Costa Rica. *J. Clean. Prod.* **2012***, 35*, 152-163.

17. Beccali, M.; Cellura, M.; Iudicello, M.; Mistretta, M. Resource consumption and environmental impacts of the agri-food sector: Life cycle assessment of Italian citrus-based products. *Environ. Manage.* **2009***, 43*, 707-724.

*18. The Australian Life Cycle Inventory Database*; http://www.auslci.com.au/index.php/Home

19. Durlinger, B.; Koukouna, E.; Broekema, R.; van Paassen, M.; Scholten, J. *Agri-footprint 4.0,* Blonk Consultants, 2017; http://www.agri-footprint.com/users/#methodology

*20. Water Use Benchmarking in the Beverage Industry,* Beverage Industry Environmental Roundtable, 2010.

21. *Towards Sustainability 2007-08.*, Australian Food and Grocery Council: Canberra, 2009.

22. Farmery, A.K. An Assessment of the Environmental Performance of Seafood and the Implications for Food Systems. PhD Thesis, University of Tasmania, Australia, 2016.

23. *NUTTAB 2010 Online Searchable Database*, Food Standards Australia New Zealand, Canberra, 2015.
